# Rectal Administration of *Leishmania* Cells Elicits a Specific, Th1-Associated IgG2a Response in Mice: New Perspectives for Mucosal Vaccination against Leishmaniasis, after the Repurposing of a Study on an Anti-Viral Vaccine Candidate

**DOI:** 10.3390/tropicalmed8080406

**Published:** 2023-08-09

**Authors:** Ilaria Varotto-Boccazzi, Sara Epis, Giulia Maria Cattaneo, Noemi Guerrini, Alessandro Manenti, Diego Rubolini, Paolo Gabrieli, Domenico Otranto, Gianvincenzo Zuccotti, Emanuele Montomoli, Claudio Bandi

**Affiliations:** 1 Department of Biosciences, University of Milan, 20133 Milan, Italy; ilaria.varotto@unimi.it (I.V.-B.); giulia.cattaneo@unimi.it (G.M.C.); paolo.gabrieli@unimi.it (P.G.); 2 Pediatric CRC ‘Fondazione Romeo ed Enrica Invernizzi’, University of Milan, 20157 Milan, Italy; gianvincenzo.zuccotti@unimi.it; 3 VisMederi, 53100 Siena, Italy; noemi.guerrini@vismederi.com (N.G.); alessandro.manenti@vismederi.com (A.M.); emanuele.montomoli@vismederi.com (E.M.); 4 Department of Life Sciences, University of Siena, 53100 Siena, Italy; 5 Department of Environmental Science and Policy, University of Milan, 20133 Milan, Italy; diego.rubolini@unimi.it; 6 Water Research Institute-National Research Council of Italy, IRSA-CNR, 20861 Brugherio, Italy; 7 Department of Veterinary Medicine, University of Bari, 70010 Valenzano, Italy; domenico.otranto@uniba.it; 8 Faculty of Veterinary Sciences, Bu-Ali Sina University, Hamedan 65175/4161, Iran; 9 Department of Biomedical and Clinical Sciences, University of Milan, 20157 Milan, Italy; 10 Department of Molecular and Developmental Medicine, University of Siena, 53100 Siena, Italy

**Keywords:** mucosal immunity, canine leishmaniasis, sera repurposing, *Leishmania* vaccine, Th1/Th2 immune polarization, LeCoVax-2

## Abstract

The mucosal immune system plays a pivotal role in the control of infections, as it represents the first line of defense against most pathogens, from respiratory viruses to intestinal parasites. Mucosal vaccination is thus regarded as a promising strategy to protect animals, including humans, from infections that are acquired by ingestion, inhalation or through the urogenital system. In addition, antigens delivered at the mucosal level can also elicit systemic immune responses. Therefore, mucosal vaccination is potentially effective also against systemic infections acquired through non-mucosal routes, for example, through the bite of hematophagous insects, as in the case of leishmaniasis, a widespread disease that affects humans and dogs. Here, we explored the potential of antigen rectal administration for the generation of anti-*Leishmania* immunity. Mice were immunized through rectal administration of whole cells of the model parasite *Leishmania tarentolae* (using a clone engineered to express the spike protein of the SARS-CoV-2 virus generated in a previous study). A specific anti-*Leishmania* IgG antibody response was detected. In addition, the recorded IgG2a/IgG1 ratio was higher than that of animals injected subcutaneously; therefore, suggesting a shift to a Th1-biased immune response. Considering the importance of a Th1 polarization as a protective response against *Leishmania* infections, we suggest that further investigation should be focused on the development of novel types of vaccines against these parasites based on rectal immunization.

## 1. Introduction

*Leishmania* spp. are a group of protozoa infecting several vertebrate species, including humans. Major diseases caused by these microorganisms comprise visceral leishmaniasis in humans and a multisystemic pathology in dogs, known as canine leishmaniasis [1,2]. Despite decades of research efforts in *Leishmania* vaccinology, none of the numerous candidate vaccines tested has been translated into products suitable to be used in large-scale vaccination campaigns in humans, and capable of determining high-level protection, associated with persistent immunity [3]. Obstacles towards developing effective *Leishmania* vaccines stem, at least in part, from the type of immunity required to achieve an effective protection against the disease [3,4]. Indeed, anti-*Leishmania* immunity should develop on the Th1 side, with classical M1 macrophage activation, and limited Th2 response, with low production of antibodies [4,5,6,7]. Indeed, an excess in non-protective antibody production in chronic leishmaniasis is associated with immune-complex disease and should ideally be avoided. In this context, adjuvants are of great importance, since molecules that polarize the response on the Th1 side are expected to favor the development of a protective response, while those biassing the response on Th2 could potentially worsen the clinical course of the disease [7,8]. Consequently, most of the investigations in *Leishmania* immunology and vaccine development have been focused on factors involved in immune-modulation, chiefly on adjuvants polarizing the response on the Th1 side. In contrast, the effects of the route of vaccine administration on the polarization of the immune response have generally been overlooked, in *Leishmania* infections as well as in other diseases. Considering the practical advantages of this type of needle-less delivery of the antigens (see Conclusion), and of the role of mucosa-associated lymphoid tissues in the modulation of both local and systemic immunity, mucosal vaccination is worthy of further investigations. Indeed, gut immunity is now regarded as a major player in immune modulation [9,10]. Vaccine administration through the enteral route could thus be exploited also for its potential to modulate the immune response on the desired direction. Here, we investigated whether rectal administration of *Leishmania* parasites is suitable to elicit a specific immune response, and whether this response develops towards the desired Th1 side. To this end, we repurposed groups of sera obtained in the context of a previous study that exploited the model parasite *Leishmania tarentolae*, as a vaccine platform in anti-viral vaccination (see details in Materials and Methods) [11,12].

## 2. Materials and Methods

### 2.1. Clones of L. tarentolae

In a previous study, *L. tarentolae* was engineered to express the spike protein antigen from SARS-CoV-2 [13]. The engineered clone of *L. tarentolae* (Lt-spike) was then used to prepare a candidate anti-COVID-19 vaccine that was tested in immunization assays in mice. The formulation used in mice immunization contained 10^8^ whole cells of the Lt-spike clone and the purified receptor binding domain (RBD) fragment, which is a portion of the spike protein. The combination of Lt-spike plus purified RDB was named LeCoVax-2. This previous study was designed to investigate the immune response of mice against SARS-CoV-2; therefore, the antibody response against *L. tarentolae* had not been determined.

### 2.2. Mice Immunization

To perform this study, we used sera samples derived from a previous experiment ([13]; authorizations: D4A18.B.1YW; D4A18.B.FX6), in which female BALB/c mice were immunized with LeCoVax-2. The animals were used according to Directive 2010/63/UE regarding the protection of animals used for experimental or other scientific purposes, enforced by the Italian Legislative Decree n° 26 of 2014 and Ministerial. Mice were rectally immunized on day 0, 21 and 35 with different formulations as follows: (i) LeCoVax-2 (Lt-spike and recombinant RBD antigen); (ii) LeCoVax-2 mixed with 10 µg Resiquimod (R848, Invivogen, San Diego, CA, USA); (iii) LeCoVax-2 mixed with 25 µg Resiquimod; (iv) PBS as placebo. Mice were also immunized subcutaneously with LeCoVax-2+ AddaVax (Invivogen, San Diego, CA, USA); sera from these mice were included in this study as a reference group, for comparison with intrarectal administration. Sera were collected on day 0 (prior to immunization), 21, 35 and 48 for the characterization of the antibody response (Table 1).

### 2.3. In-House Enzyme-Linked Immunosorbent Assay (ELISA)

#### 2.3.1. Preparation of *Leishmania tarentolae* Antigen (LtAg)

*L. tarentolae* (P10 strain) promastigotes were cultured in brain heart infusion (BHI) liquid medium supplemented with 5 μg/mL porcine hemin, 50,000 U/L penicillin, 50 mg/L streptomycin (Jena Bioscience, Jena, Germany) at 26 °C in the dark under aerated conditions. *L. tarentolae* promastigote antigens (LtAg) were prepared following the protocol described in [14]. Briefly, *L. tarentolae* cells were washed twice in phosphate buffered saline (PBS), then the pellet was lysed with a buffer containing 50 mM Tris, 5 mM EDTA and protease inhibitors (Invitrogen, Waltham, MA, USA) and then subjected to three rapid freeze/thaw cycles followed by six sonication pulses of 20 s/40 W. Then, the samples were centrifuged at 10,000× *g* for 20 min at 4 °C and the supernatants were collected and stored at −80 °C until use. The protein concentration was determined with the Nanodrop spectrophotometer.

#### 2.3.2. Set Up of In-House Leishmania IgG ELISA Assay

An in-house IgG ELISA assay was set up using LtAg as the coating antigen for the detection of specific *Leishmania* antibodies in the sera of murine samples. A series of preliminary assays was performed to select the optimal antigen concentration (0.5, 1, 1.5, 3, 4 μg/mL) testing five murine sera collected from BALB/c mice immunized via subcutaneous route with *L. tarentolae* wild type promastigotes (strain P10) and obtained with the preliminary study described in [13]; these sera were previously tested in immunofluorescent assay and resulted positive for *Leishmania* antibody response. Plates were coated with the five different LtAg concentrations and incubated overnight at 4 °C. After three washes with tris-buffered saline (TBS)–0.05% (*v*/*v*) Tween 20 (TBS-T), plates were blocked for 1 h with TBS-T containing 5% (*w*/*v*) non-fat dry milk (NFDM; Euroclone, Pero, Italy). Subsequently, two-fold serial dilutions, starting from 1:100 in blocking buffer, were performed and then 100 μL of each serial dilution was added for 1 h at 37 °C. Then, goat anti-mouse IgG1 HRP-conjugated (Bethyl Laboratories, Montgomery, TX, USA) diluted at 1:50,000 was added for 30 min at 37 °C. Finally, 3,3′,5,5′-tetramethylbenzidine substrate (Sigma Aldrich, St. Louis, MO, USA) was added for 20 min and then HCl solution 0.5 N to stop the reaction (Fisher Chemical, Waltham, MA, USA).

### 2.4. Detection of Leishmania IgG1 and IgG2a in Murine Sera

The four animal groups and the control group are presented in Table 1. Sera collected from each animal and at each time point were tested in ELISA assay to investigate the presence of specific IgG *Leishmania* antibodies. The assay was performed as described above according to the following optimized parameters: 3 μg/mL of LtAg as coating concentration, isotype-specific HRP-conjugated secondary antibodies diluted both 1:50,000 (goat anti-mouse IgG1, Bethyl Laboratories, Montgomery, TX, USA or goat anti-mouse IgG2a, Abcam, Cambridge, UK) and sera two-folded diluted starting from 1:50 in blocking buffer. A cut-off value was obtained for each plate by multiplying by three the average of the blank optical density (OD) signal derived from wells not containing the analyte (background) [15].

### 2.5. Statistical Analysis

Variation in IgG1 and IgG2a levels among the five experimental treatments across the three time points (day 21, 35 and 48 post-vaccination) was assessed by means of gamma generalized linear mixed models (GLMMs) with log-link function, including experimental (5-level fixed factor; see Table 1), time point (3-level fixed factor) and their interaction as predictors. Mouse identity was included as a random intercept effect. Fitted models accounted for heterogeneity of variances in IgG values between time points (IgG1) and experimental groups (IgG2a). To assess variation in Th1 polarization among vaccinated groups of mice, we fitted a linear model of the IgG2a/IgG1 ratio at day 48 post-vaccination including the experimental group as a 4-level factor (control mice excluded). Post hoc pairwise comparisons were computed to investigate differences in IgG levels or IgG2a/IgG1 ratio between experimental groups (within each time point for gamma GLMMs of IgG1 and IgG2a), adjusting *p*-values according to the Tukey method (on families of 5 or 4 estimates, respectively). The association between anti-*Leishmania* IgG1 and anti-RBD IgG1 antibody levels assayed in the same individuals (n = 40) was assessed by the Spearman correlation coefficient. Gamma GLMMs were fitted using the glmmTMB R package (ver. 1.1.3). Pairwise comparisons were performed using the emmeans and multcomp R packages (ver. 1.7.1 and 1.4, respectively). All statistical analyses were performed using the R software (ver. 4.0.4; [16]).

## 3. Results and Discussion

The purpose of the current study was to explore whether rectal administration of whole, inactivated cells from *L. tarentolae* is suitable to induce a specific antibody response against *L. tarentolae* itself. To perform this study, we took advantage from a previous study, in which whole cells of *L. tarentolae* (from the clone Lt-spike) had been administered to BALB/c mice through the rectal route, with the purpose of assaying a candidate vaccine against COVID-19 (see [13] and Material and Methods section). The aim of this previous study was to determine the response against SARS-CoV-2; determination of anti-*Leishmania* antibody production was thus not performed. Sera from this previous work had been stored at -80 C; we thus retrieved the sera and determined the presence of antibodies against *L. tarentolae* using an in-house ELISA assay, also typing the two main IgG subclasses, IgG1 and IgG2a.

### 3.1. Set Up of In-House ELISA Assay

Lt-Ag, the antigen prepared from *L. tarentolae* promastigotes, was tested for its ability to detect specific *Leishmania* IgG antibodies in the sera of immunized mice. Of the four antigen concentrations assayed, we finally chose a concentration of 3 μg/mL for the coating of ELISA plates. Among the five positive sera used to set up the ELISA assay, the serum with the highest OD value at the first dilution was chosen as positive control to be used in the following test (Tables S1 and S2).

### 3.2. Anti-Leishmania IgG Antibody Response in Mice after Rectal Immunization, and Correlation with the IgG Response against SARS-CoV-2

**Figure 1:**
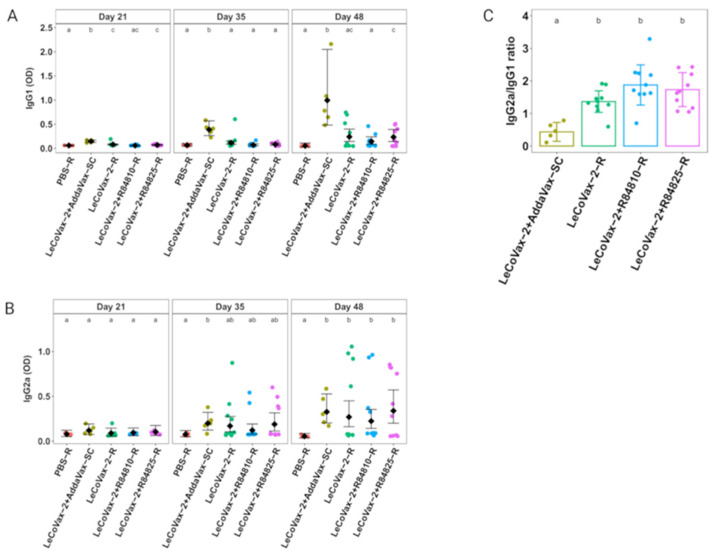
IgG1 and IgG2a antibody response after rectal immunization with LeCoVax-2. (**A**,**B**) Variation of IgG1 and IgG2a levels detected at days 21, 35 and 48 after subcutaneous or rectal administration of different formulations of LeCoVax-2 in mice (n = 10 mice per experimental group, except for PBS-R, n = 5). Dots represent original data points; diamonds represent estimated mean values (with 95% CI) from gamma GLMMs accounting for heterogeneity of variances (see Section 2). (**C**) IgG2a/IgG1 ratio at day 48 after subcutaneous or rectal administration of the different formulations in mice (bars represent mean values; error bars represent standard deviation). In all plots, in the upper part of each graph, different lowercase letters indicate significant (*p* < 0.05) differences at post hoc tests (after Tukey correction for multiple testing) (within each time point for (**A**,**B**)). For example, label ‘ac’ denotes a statistically significant difference compared to groups labeled as ‘b’, but a non-significant difference compared to groups ‘a’ and ‘c’.

Table 1 presents the groups and animals, and the details of the preparations used in immunization. The IgG1 and IgG2a responses against *L. tarentolae* are reported in Figure 1. IgG1 values showed a statistically significant differential variation across time points among different formulations (gamma GLMM, treatment × time point interaction, χ2 = 35.8, d.f. = 8, *p* < 0.0001), with an increase of OD values mostly after the second administration (Figure 1A). After 21 days, mice vaccinated subcutaneously with LeCoVax-2 plus AddaVax showed significantly higher levels of IgG1 antibodies compared to other vaccinated mice and controls (*p* < 0.001 for all comparisons). The same trend was observed after 35 days, while after the third administration (i.e., after day 48), a higher number of positive mice was scored in all experimental groups (Table 1). As reported in Table 1, mice immunized subcutaneously with LeCoVax-2 plus AddaVax developed anti-*Leishmania* antibodies with statistically significant differences compared to the control group (*p* < 0.0001), as well as to groups that received intrarectal vaccination, with LeCoVax-2 (*p* < 0.05), LeCoVax-2 + R84810 (*p* < 0.001) and LeCoVax-2 + R84825 (*p* < 0.05). In addition, mice immunized through the rectal route with LeCoVax-2 + R84825 also showed statistically significant differences compared to the controls (*p* < 0.05) (Figure 1A). Interestingly, mice that showed antibodies against *Leishmania* were the same that produced antibodies against SARS-CoV-2 in the previous study (Table 1) [13], except for two animals (from the subcutaneous group) that developed only antibodies against *Leishmania*. Overall, a positive correlation between the two responses (i.e., against SARS-CoV-2 and *Leishmania* spp.) was observed (Spearman’s rank correlation ⍴ = 0.70; *p* < 0.0001). This positive correlation suggests that variability of the response of mice in the groups intrarectally immunized may be due to the characteristics of the route of administration. As discussed in [13], rectal vaccination implies that the retention of the antigen in the intestine is variable, in some way unpredictable, and this can explain the variability of the results obtained after vaccination through this route.

### 3.3. Specific IgG Subtype Responses for the Different Routes of Administration

The specific IgG2a immune response against *Leishmania* was assessed by ELISA assay and the IgG2a/IgG1 ratio was then calculated. A statistically significant differential variation of IgG2a across the three time points among different formulations (gamma GLMM, treatment × time point interaction, χ2 = 87.7, d.f. = 8, *p* < 0.0001) was found, with an increase of OD values starting from the second dose of the treatment (day 35). After the first immunization, no significant differences were detected in the groups, while after 35 days, an increasing number of positive animals in all immunized groups was recorded, showing a statistically significant difference between mice vaccinated with LeCoVax-2 + AddaVax and mice of the control group (*p* < 0.05) (Figure 1B). After the third administration (48 days), the highest number of positive mice was detected in all groups, with statistically significant differences with the PBS control group (for all comparisons *p* < 0.0001 except for LeCoVax-2 and LeCoVax-2 + R84810: *p* < 0.001) (Figure 1B).

The IgG2a/IgG1 ratio, a marker associated with the polarization of the immune response on the Th1 or Th2 sides [17], revealed that mice receiving a subcutaneous administration showed a IgG2a/IgG1 ratio lower than 1 (value = 0.43), suggesting a shift towards a Th2 response; conversely, mice intrarectally immunized showed a higher IgG2a/IgG1 ratio, over 1.5, indicating a Th1-biased immune response (linear model, effect of treatment group: χ2 = 37.94, d.f. = 3, *p* < 0.0001). The differences were statistically significant for all three groups intrarectally immunized compared with subcutaneously immunized mice (LeCoVax-2: *p* < 0.01; LeCoVax-2 + R84810: *p* < 0.0001; LeCoVax-2 + R84825: *p* < 0.001) (Figure 1C). These results agree with those of previous studies on other infectious agents, showing that the subcutaneous route of antigen administration is more likely to induce a Th2 immune response compared with rectal administration, which is commonly associated with a more balanced response, or with a shift on the Th1 side [18,19,20]. The highest IgG2a/IgG1 ratio was observed in mice intrarectally immunized with LeCoVax-2 with the lower dose of adjuvant (value = 1.9). Furthermore, intrarectal administration of LeCoVax-2 determined a Th1-biased response against *L. tarentolae* also in the absence of any adjuvant. The possibility to induce a correctly polarized immune response against *Leishmania* spp., in the absence of adjuvant, further emphasizes the potential of rectal vaccination for developing effective leishmaniasis vaccines.

## 4. Conclusions

Enteral vaccination presents both drawbacks and advantages compared to other modes of antigen delivery [21]. A first advantage is that enteral vaccination, similarly to other forms of mucosal vaccinations, is potentially suitable to elicit an immune response at the mucosal level, with production of secretory IgA antibodies. A second advantage is the possibility to exploit the modulation of the immune response, which is one of the functions of gut-associated lymphoid tissues (GALT). Indeed, a major role of GALT is the interplay with food-derived molecules and with the microbiota, in the modulation and balancing of the overall functioning of the immune system [9,10]. Finally, preparation of vaccines for enteral vaccination is facilitated by the lower requirements in terms of purity and sterility, compared with classic vaccines, e.g., those for intramuscular administration. So far, a sole study investigated the potential of enteral vaccination against *Leishmania*, using a murine model. In this study, a cell lysate of *Leishmania amazonensis* was administered orally; parameters of the immune modulation were then determined, with evidence for a specific, Th1-biased polarization of the response [22]. Besides this study, which dates to 2003, all successive investigations on mucosal vaccination in *Leishmania* spp. have exploited the nasal/inhalation route for the administration of the antigen (e.g., [23,24]). Overall, the results of our investigation on rectal administration of *Leishmania* antigens are coherent with those obtained after oral administration in the 2003 study, displaying a significant production of IgG antibodies against the administered *Leishmania* species, with a shift toward a specific Th1-associated IgG2a response. In conclusion, although in the present study we used an engineered strain of *L. tarentolae,* and this could have influenced the specific production of anti-*Leishmania* antibodies, considering the importance of Th1 polarization for a protective response against *Leishmania* spp. infections, and the advantages of mucosal vaccination, rectal immunization is worth of further investigation towards the development of novel vaccines against these parasites.

## 5. Patents

The antigen Lt-spike and its potential application in vaccination against coronaviruses have been described in the PCT/IB2022/051585 (23 February 2022). The antigen Lt-RBD and its potential application have been described in patent application no. IT 02021000004160 (23 February 2021).

## Figures and Tables

**Table 1:** Details of experimental groups and of IgG-positive samples at day 48 in each tested group.

Experimental Group	Formulation	IgG1 *Leishmania* Positive Samples	IgG1 SARS-CoV-2 Positive Samples	IgG2a *Leishmania* Positive Samples
*Subcutaneous injection*				
LeCoVax-2 + AddaVax-SC	2 × 10^7^ cells Lt-spike + 10 μg RBD + AddaVax	5/5	3/5	5/5
*Rectal administration*				
PBS-R	PBS solution (control)	0/5	0/5	0/5
LeCoVax-2-R	1 × 10^8^ cells Lt-spike + 20 μg RBD	4/10	4/10	4/10
LeCoVax-2 + R848 10-R	1× 10^8^ cells Lt-spike + 20 μg RBD + 10 μg R848	2/10	4/10	4/10
LeCoVax-2 + R848 25-R	1 × 10^8^ cells Lt-spike + 20 μg RBD + 25 μg R848	4/10	6/10	6/10

Lt: *Leishmania tarentolae*; RBD: Receptor Binding Domain; SC: subcutaneous; R: rectal; AddaVax: squalene-based oil-in-water nano-emulsion; R848: Resiquimod.

## Data Availability

The datasets supporting the findings of this article are included within the article and its Supplementary Materials.

## Supplementary Materials

The following supporting information can be downloaded at: https://www.mdpi.com/article/10.3390/tropicalmed8080406/s1, Table S1. Plate layout and OD values of the set-up of the first ELISA assay. Table S2. Plate layout and OD values of the second set-up of the first ELISA assay.
